# Evaluation of the impact of acidic medications and fluoride-containing mouthwash on the enamel surface using quantitative light-induced fluorescence, microhardness, and scanning electron microscopy: an in vitro study

**DOI:** 10.1186/s12903-025-05523-3

**Published:** 2025-01-27

**Authors:** Saanya Bhasin, Simran Singh, Manuel Sebastian Thomas, Karuna Yarmunja Mahabala, Ramya Shenoy

**Affiliations:** 1 Manipal College of Dental Sciences Mangalore, Manipal Academy of Higher Education, Karnataka, Manipal 576104 India; 2 Department of Conservative Dentistry and Endodontics, Manipal College of Dental Sciences Mangalore, Manipal Academy of Higher Education, Karnataka, Manipal 576104 India; 3 Department of Pediatric and Preventive Dentistry, Manipal College of Dental Sciences, Mangalore, Manipal Academy of Higher Education, Karnataka, Manipal 576104 India; 4 Department of Public Health Dentistry, Manipal College of Dental Sciences, Manipal Academy of Higher Education, Mangalore, Karnataka, Manipal 576104 India; 5 Department of Conservative Dentistry and Endodontics, Manipal College of Dental Sciences Mangalore, Manipal Academy of Higher Education, Manipal, Karnataka 576104 India

**Keywords:** Anti-asthmatic, Dental erosion, Dentistry, Enamel, Fluoride mouthwash, Hardness, Medication, Vitamin C

## Abstract

**Background:**

Due to their acidic nature, certain medications can have deleterious effects on tooth enamel. Fluoride is a popular method for reversing these effects. Therefore, this study aimed to assess the impact of acidic medications, specifically anti-asthmatic drugs and vitamin C tablets, on enamel surfaces and to investigate the effects of fluoride following drug exposure.

**Methods:**

An in vitro experimental study was conducted on ninety-six healthy human posterior teeth. Forty-eight samples were tested for surface demineralization via quantitative laser fluorescence (QLF), and the other forty-eight samples were tested for enamel microhardness via a Vickers hardness tester. The samples were divided into six groups: (i) Group DW_NF- samples immersed in distilled water with no fluoride exposure; (ii) Group DW_F- samples immersed in distilled water with intermittent fluoride exposure; (iii) Group VC_NF- samples treated with vitamin C only; (iv) Group VC_F- samples treated with vitamin C and fluoride mouthwash; (v) Group SB_NF- samples exposed to salbutamol inhaler with no fluoride exposure; (vi) Group SB_NF- samples exposed to salbutamol inhaler followed by fluoride exposure. For the evaluation of surface morphology by scanning electron microscopy (SEM), two random samples used for QLF from each group were taken. The Wilcoxon signed-rank test, Kruskal‒Wallis test, and post hoc test were applied as appropriate. The *p* value was set at 0.05.

**Results:**

Following exposure to medication, significantly higher QLF values were observed in Group VC_F and Group SB_F than in Group DW-NF (control group) (*p* = 0.15 and 0.004, respectively). The difference in the QLF values was significantly greater in Group VC_NF than in the control group DW-NF, indicating greater demineralization with acidic medications (*p* = 0.034). Significant changes in surface microhardness were detected in Group VC_F compared with the control group (*p* = 0.024). Qualitative analysis of SEM images revealed erosion at the enamel surface in all groups except the control group, with the most prominent erosion in the vitamin C- and fluoride-treated groups (VC_F group).

**Conclusion:**

This study demonstrated the erosive potential of vitamin C tablets on enamel surfaces. Furthermore, the use of acidic fluoride mouthwash immediately after exposure to acidic medication exacerbates enamel demineralization.

## Introduction

The gradual loss of mineralized tooth structure from exposure to acids without the involvement of oral bacteria is called tooth erosion [1]. In the direct presence of acids, enamel demineralizes [2]. These acids can originate from within the body or externally [3]. Many drugs or agents have the potential to erode tooth enamel because of their acidic nature [4]. For chronic illness, medications are usually administered for a long duration; thus, prolonged exposure to acidic medications may lead to tooth erosion [5].

Asthma is a significant noncommunicable disease that affects individuals of all ages. In 2019, it affected an estimated 262 million people. Inhaled medications are essential for managing symptoms and supporting individuals in leading normal, active lives [6]. The relationship between asthma and dental erosion has been debated over the years. Some studies support the link between dental erosion and asthma medications and suggest prophylactic measures for asthmatic patients [7, 8]. Another study, however, concluded that there is no clear association between asthma medications and tooth erosion [9]. The acidity of some antiasthmatic drugs is considered a contributing factor in studies that support this connection [10, 11]. Salbutamol is a beta-2 agonist that is routinely used in the management of chronic bronchospasm at all ages. pH values lower than 5.5 are the critical value required for hydroxyapatite dissolution, especially when it is in powdered form [12–14].

Vitamin C (L-ascorbic acid) is available in various forms, including syrups, chewable tablets, capsules, and effervescent powders. Owing to its low pH, vitamin C, especially its chewable form, has a detrimental effect on tooth surfaces [15]. The popularity of vitamin C as a health supplement has increased in recent years, particularly due to the pandemic [16]. Therefore, understanding its effects on dental enamel is crucial for recommending preventive measures to individuals who use it regularly.

Enamel erosion starts as surface demineralization initially and then continues to soften and dissolve the enamel substance [17]. Fluoride therapy is one of the most commonly used treatment modalities for the remineralization of teeth [18]. Therefore, this in vitro study aimed to evaluate the effects of acidic medicaments such as an anti-asthmatic medication (Asthalin (Salbutamol) Inhaler, Cipla Ltd., Mumbai, India) and a vitamin C chewable tablet (Limcee 500 mg, Abbott Health Care Pvt Ltd, Bangalore, India) and the effects of the immediate use of acidic fluoride mouthwash (Colgate MaxFresh Plax Peppermint Fresh Mouthwash, Colgate^®^, Mumbai, India) on the enamel surface. The null hypothesis tested was that acidic medications and fluoride application would have no effect on enamel demineralization/remineralization, microhardness, or surface topography compared with those of the control group.

## Materials

This in vitro experimental study was conducted on extracted healthy human permanent posterior teeth after receiving approval from the Institutional Ethics Committee (IEC/MCODSM/22052). Consent was obtained from all patients for their teeth to be used for research purposes during the time of extraction.

### Sample size calculation

The sample size calculation for two means was performed using the following formula: *n* = 2[(Z_(1-α/2) + Z_(1-β)]^2 σ^2)/d^2. In this study, the Z value for a 1% alpha error was set at 2.58, and the Z value corresponding to 95% power was set at 1.64. The standard deviations (σ) used for the calculation were derived from Table 1 of the study by Favaro et al. [19], with values of 1.54 for the control group and 5.08 for the fluoride mouthwash group, resulting in an average standard deviation of 3.31 units. Considering a clinically significant difference (d) of 7 units, the required sample size (n) was determined to be 8 per group.

**Table 1:** Pre- and postexposure evaluation of quantitative laser fluorescence (QLF) and microhardness

Groups^#^	Timeline	Mean (SD)	Median (IQR)	*p* value*
**Quantitative Laser Fluorescence (QLF value)**				
DW_NF	Baseline	0.5 (1.07)	0 (0,0.75)	**0.011**
	Final	1.63 (0.92)	1 (1,2.75)	
DW_F	Baseline	1.25 (0.89)	1.5 (0.25,2)	**0.026**
	Final	3.63 (2.50)	3 (2,4.75)	
VC_NF	Baseline	0.38 (1.06)	0 (0,0)	**0.011**
	Final	4.25 (2.12)	4 (2.25,5.75)	
VC_F	Baseline	2.63 (2.2)	3 (0.25,4)	**0.017**
	Final	4.5 (1.51)	4.5 (3,5.75)	
SB_NF	Baseline	0.75 (1.17)	0 (0,1.75)	**0.011**
	Final	3.5 (1.31)	3.5 (2.25,4)	
SB_F	Baseline	1.63 (2.83)	0 (0,2.75)	**0.011**
	Final	5 (2)	5 (4,5.75)	
**Microhardness (Vickers’ Harness Number)**				
DW_NF	Baseline	363.74 (72.55)	355.30 (292.95,432.75)	0.779
	Final	339.98(62.01)	326.45 (285.33,394.65)	
DW_F	Baseline	337.66(58.41)	351.45 (297.60,365.98)	0.779
	Final	341.06(77.92)	339.50 (296.18,376.55)	
VC_NF	Baseline	350.28 (49.42)	330.10 (311.35,379.13)	0.401
	Final	318.20 (50.72)	318.60 (289.83,345.40)	
VC_F	Baseline	392.49 (87.15)	373 (312.63,473.03)	**0.036**
	Final	316.50 (32.49)	316.65 (294.98,347.95)	
SB_NF	Baseline	405.64 (120.88)	343.55 (307.78,545.55)	0.069
	Final	323.26(33.51)	314.30 (292.25,359.18)	
SB_F	Baseline	354.24 (51.83)	349.30 (313.78,409.55)	0.123
	Final	320.36 (40.93)	327.90 (318.48,347.20)	

# DW- Distilled water; Vitamin C (VC); Salbutamol (SB); No fluoride exposure (NF); Fluoride exposure (F); The sample size for each group was 8 (*n* = 8)

* A *p*-value less than 0.05 denotes statistical significance

### Sample selection, sectioning, and storage

Forty-eight recently extracted impacted third molars with intact anatomical crowns were used for the study. These teeth were cleaned and polished to remove any surface debris or other contaminants. Those showing no evidence of enamel cracks, carious lesions, or white spot lesions were included as samples. The samples with values greater than seven according to quantitative laser fluorescence (QLF) (DIAGNOdent^®^ pen, Kavo, Biberach Germany) were excluded from the study because they suggest surface demineralization [20]. The teeth were initially sectioned at the cementoenamel junction (CEJ), and the coronal portions were then sliced mesiodistally into buccal and lingual halves via a low-speed diamond disc (Diamond Disk DA0001 4 A, Toboom, India), resulting in 96 tooth half-sections. To prevent bacterial or fungal growth, the selected teeth and the prepared samples were subsequently stored in 0.1% thymol solution until use.

Forty-eight sections were selected for evaluation of the demineralization/remineralization effect via QLF [21–23]. An acid-resistant nail varnish was applied to all the surfaces of these samples leaving a window of 4 × 4 mm in the middle of each tooth for demineralization/ remineralization assessment. The remaining forty-eight samples were used for assessing the surface microhardness. These samples were encased in an acrylic resin cylinder, exposing the enamel surface. The samples were subsequently ground under running water using a 600- and 1200-grit silicon-carbide paper polishing machine to achieve a flat surface [24].

### Medicaments used and application

The details of the medicaments used for this study are provided in Table 2. The six study groups in the present study were as follows:

**Table 2:** Details of the medicaments used in the study

Medicament	Company details	Composition
Chewable Vitamin C tablet	Limcee 500 mg, Abbott Health Care Pvt Ltd, Bangalore, India	Each uncoated chewable tablet contains Ascorbic Acid IP 100 mg ; Sodium Ascorbate IP 450 mg (eq.to Ascorbic Acid 400 mg); Excipients (q.s.); Sunset yellow FCF (coloring agent)
Salbutamol inhaler	Asthalin inhaler IP 100 Mcg/Dose, Cipla Ltd., Mumbai, India	Each actuation delivers Salbutamol sulfate IP (equivalent to 100mcg), suspended in propellant HFA 134a (q.s.)
Fluoride mouthwash	Colgate Maxfresh peppermint fresh mouthwash, Colgate^®^ Plax^®^ product, Colgate^®^, Mumbai, India	Aqua Glycerin, Propylene Glycol, Sorbitol (humectants); (Cetylpyridinium Chloride (CPC) (0.05–0.1% - antibacterial agent); Sodium Fluoride (225ppm - anticariogenic agent); Others- Poloxamer 407 (emulsifier); Potassium Sorbate (preservative); Menthol and flavoring agents; CI 42051 (Colorant):

(i) Group DW-NF: Enamel samples were immersed in distilled water (DW) with no fluoride (NF) exposure (control group).
(ii) Group DW_F: Enamel samples were immersed in distilled water (DW) with intermittent fluoride (F) exposure. The samples were immersed in fluoride mouthwash for 1 min, twice daily, for 14 days.
(iii) Group VC_ NF: Enamel treated with vitamin C (VC) followed by NF exposure. Chewable VC was dissolved in 1 mL of distilled water, and the paste was applied to the enamel surface for 1 min. This treatment was repeated twice daily for 14 days.
(iv) Group VC_ F: Enamel treated with vitamin C (VC) followed by fluoride (F) exposure. The treatment was the same as that described above, with the addition of immediate exposure to fluoride mouthwash for 1 min. This process was repeated twice daily for 14 days.
(v) Group SB_NF: Samples treated with a salbutamol inhaler (SB) without NF exposure: Two puffs from the inhaler were expressed onto each tooth and were kept undisturbed on the enamel surface for 1 min. This procedure was repeated twice daily for 14 days.
(vi) Group SB_F: Samples treated with a salbutamol inhaler (SB) with F exposure. The treatment was the same as described above, with the addition of immediate exposure to fluoride mouthwash for 1 min. This process was repeated twice daily for 14 days.

### Recording of pH

The pH of each medication was assessed and recorded by a digital pH meter (Manti Lab solution, India) connected to an electrode calibrated with standard solutions of pH 4.0 and 9.0 [22]. The vitamin C tablet was dissolved in 100 ml of distilled water and then subjected to pH evaluation. The fluoride mouthwash was placed in a beaker, and the pH was recorded similarly. To test the pH value of salbutamol, ten aerosol bursts of the medicine were introduced into 100 ml of distilled water.

### Assessment of demineralization/remineralization

Forty-eight samples that were allocated to assess demineralization/remineralization after exposure to medications were assigned via simple randomization to the six groups mentioned above equally (*n* = 8). The samples were scanned via quantitative light fluorescence (DIAGNOdent^®^ pen - Kavo, Biberach, Germany). The readings were recorded both at baseline and after 2 weeks of enamel surface treatment with the respective medications. A proximal fissure probe was inserted into the DIAGNOdent pen, and the pen was calibrated according to the DIAGNOdent^®^ pen user manual. The distal end of the tip was placed vertically into the depression at the center of the ceramic reference until the signal tone stopped, which ensured that the calibration was complete. The samples were then tested by positioning the tip in close contact with the tooth surface. The tip was run around the measuring site to capture fluorescence from all angles. The diagnodent pen detects demineralization in cleaned teeth by inducing fluorescence with laser light [21].

### Assessment of surface microhardness

The forty-eight teeth enamel samples allocated for microhardness testing were equally distributed among the six study groups (*n* = 8). Baseline microhardness readings were evaluated via a Vickers hardness tester (Mitutoyo, Kanagawa, Japan). The indenter had a load of 100 g and a dwell time of 15 s. To visualize and precisely measure the diagonals in the diamond-shaped indentations, an optical microscope with image analysis software (AVPAK 20, Mitutoyo, Tokyo, Japan) was used. On the basis of the mean length of the diagonal, microhardness values were calculated and converted into Vickers hardness numbers (VHN1). Following exposure of the samples to different treatments, the same tests were repeated after 2 weeks (VHN2). The percentage of loss from the surface microhardness was calculated via the Vickers microhardness number (%VHN) following acid exposure using the formula: 100× (VHN2-VHN1) ⁄ VHN1 [23].

### Assessment of surface morphology

Two random samples from each group were selected after noninvasive DIAGNOdent^®^ measurements and sent for scanning electron microscopy (SEM) analysis. The samples were mounted on brass rings via nonconductive carbon tape and then placed in a sputtering and carbon cord evaporation machine (Q150R Plus ES, Quorum, East Sussex, United Kingdom) for 30 min. A layer of gold (4 nm) was sputtered onto the samples under an argon atmosphere. SEM (Carl Zeiss Supra 55 FESEM, Germany) was subsequently conducted at 2000× magnification on the same samples. Qualitative observations were made to assess surface topographic changes [25].

### Statistical analysis

Data analysis was carried out using SPSS (version 20.0) (IBM SPSS^®^ Statistics) -Java (TM) Platform SE binary-IBM Corp: London: UK (Trial Version). The descriptive statistics were tabulated. The normality of the data was checked using the Shapiro‒Wilk test. The Wilcoxon signed-rank test was applied for intragroup comparisons, and the Kruskal‒Wallis test followed by Dunn’s post hoc test was applied for intergroup comparisons. The *p* value was set at 0.05 to assess statistical significance.

## Results

### pH evaluation

The most acidic pH was found to be that of vitamin C (4.5–4.65), followed by fluoride (5.2–5.43), salbutamol (5.2–6.0), and distilled water (6–6.05).

### Quantitative laser fluorescence (QLF) evaluation

The QLF data pre- and postexposure to medications were tabulated, and the same statistical analysis was performed (Tables 1 and 3). As shown in Table 1, the intergroup pretreatment and postexposure values were statistically significant in all the subgroups (*p* < 0.05). As depicted in Table 3, the baseline QLF readings were not different between the samples tested (*p* = 0.074). After the final treatment, the salbutamol with fluoride (SB_F) group presented the highest mean and standard deviation (5.0 ± 2.0), followed by the vitamin C with fluoride (VC_F) group (4.5 ± 1.51). These values were statistically significant compared with those of the control group (DW_NF) (1.63 ± 0.92), with *p* values of 0.015 and 0.004, respectively. For differences in the QLF values (ΔQLF), vitamin C (VC_NF) (3.88 ± 2.23) had the highest mean and standard deviation, followed by salbutamol with fluoride (SB_F) (3.38 ± 1.77) (Table 3). Post hoc analysis of ΔQLF revealed a statistically significant difference between the DW_NF and VC_NF groups (*p* = 0.034).

**Table 3:** Quantitative laser fluorescence (QLF) data before and after treatment within each timeline

Timeline	Groups^#^	Mean (SD)*	*p* value
Baseline (Before treatment)	DW_NF	0.5 (1.07) ^a^	0.074
	DW_F	1.25 (0.89) ^a^	
	VC_NF	0.38 (1.06) ^a^	
	VC_F	2.63 (2.2) ^a^	
	SB_NF	0.75 (1.17) ^a^	
	SB_F	1.63 (2.83) ^a^	
Final (After treatment)	DW_NF	1.63 (0.92) ^a^	**0.005**
	DW_F	3.63 (2.5) ^a, b^	
	VC_NF	4.25 (2.12) ^a, b^	
	VC_F	4.5 (1.51) ^b^	
	SB_NF	3.5 (1.31) ^a, b^	
	SB_F	5 (2) ^b^	
Difference in QLF (ΔQLF)	DW_NF	1.13 (0.84) ^a^	**0.023**
	DW_F	2.38 (2.2) ^a, b^	
	VC_NF	3.88 (2.23) ^b^	
	VC_F	1.88 (1.13) ^a, b^	
	SB_NF	2.75(1.28) ^a, b^	
	SB_F	3.38 (1.77) ^a, b^	

# DW- Distilled water; Vitamin C (VC); Salbutamol (SB); No fluoride exposure (NF); Fluoride exposure (F)

* In the Mean (± SD) values of each timeline, different superscript letters indicate statistically significant differences (*p* < 0.05)

### Surface hardness evaluation

A statistically significant reduction in enamel microhardness from pre- to posttreatment was observed only in the group treated with vitamin C followed by fluoride (VC_F) (*p* = 0.036) (Table 1). Both the baseline (pretreatment) values and the posttreatment surface hardness values did not significantly differ between the groups (*p* > 0.05) (Table 4). The VC_F group presented the greatest mean absolute change in the percentage of surface hardness (20.8 ± 11.28 VHN), which was significantly greater than that of the control group (DW_NF) (5.88 ± 13.88 VHN) (*p* = 0.024).

**Table 4:** Changes in the enamel surface upon exposure to various medicaments

Timeline	Groups^#^	Mean (SD)*	*p* value
Baseline Hardness	DW_NF	363.74 (72.55) ^a^	0.93
	DW_F	337.66 (58.41) ^a^	
	VC_NF	350.28 (49.42) ^a^	
	VC_F	392.49 (87.15) ^a^	
	SB_NF	405.64 (120.88) ^a^	
	SB_F	354.24 (51.83) ^a^	
Final Hardness	DW_NF	339.98 (62.01) ^a^	0.939
	DW_F	341.06 (77.92) ^a^	
	VC_NF	318.2 (50.72) ^a^	
	VC_F	316.5 (32.49) ^a^	
	SB_NF	323.26(33.51) ^a^	
	SB_F	320.36 (40.93) ^a^	
Absolute change in percentages*	DW_NF	5.88 (13.88) ^a^	**0.038**
	DW_F	18.62 (16.49) ^a, b^	
	VC_NF	16.62 (15.57) ^a, b^	
	VC_F	20.8 (11.28) ^b^	
	SB_NF	18.01 (19.49) ^a, b^	
	SB_F	14.98 (9.28) ^a, b^	

# DW- Distilled water; Vitamin C (VC); Salbutamol (SB); No fluoride exposure (NF); Fluoride exposure (F)

* In the Mean (± SD) values of each timeline, different superscript letters indicate statistically significant differences (*p* < 0.05)

### Scanning electron microscopy evaluation

**Fig. 1:**
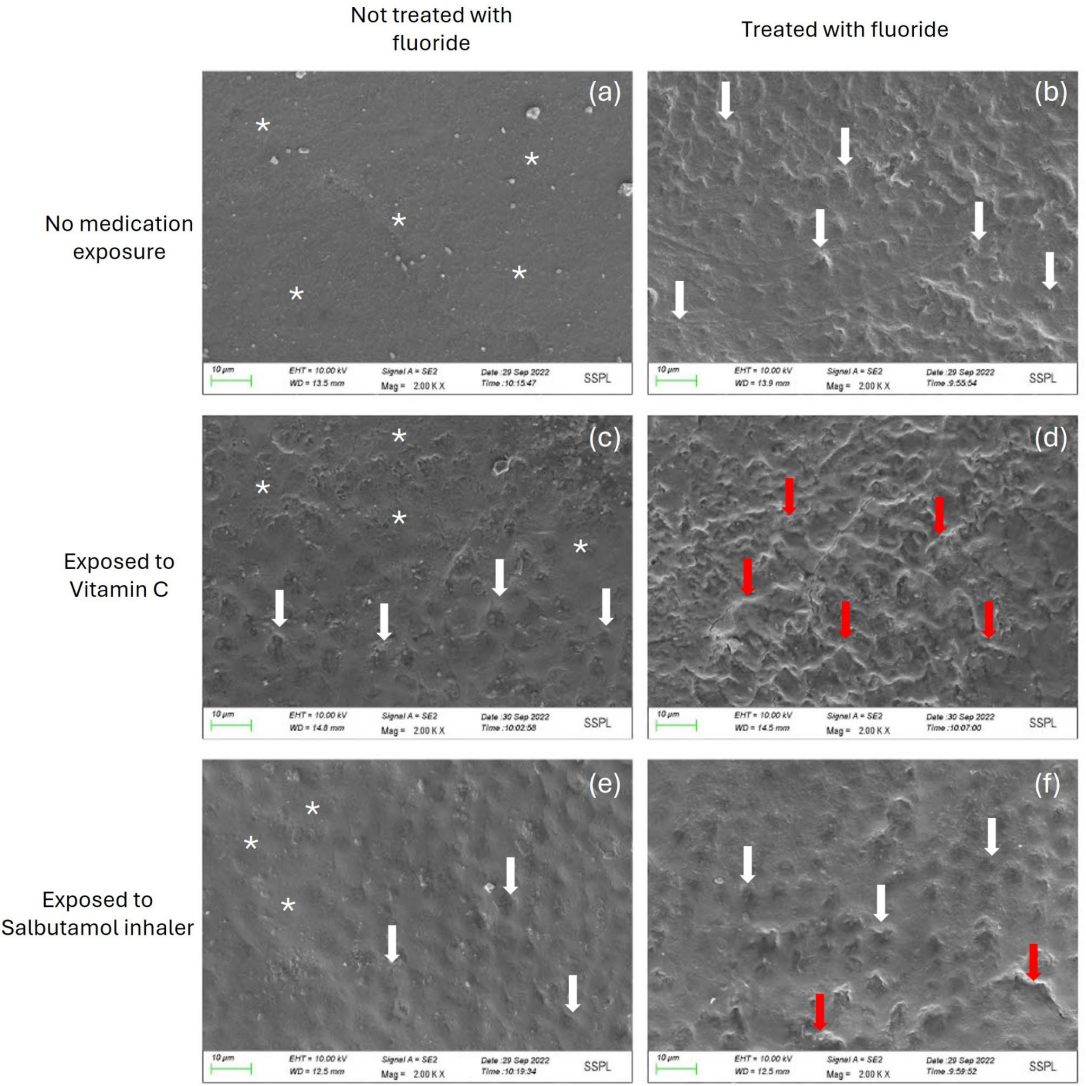
(**a-f**): Scanning electron microscopy images of the sound enamel surface after exposure to medications: (**a**) Distilled water only (control group: DW_NF) shows a smooth surface (white asterisk), (**b**) fluoride (DW_F) shows the removal of the peripheral regions of the prisms, leaving relatively unaffected prism cores (white arrow), (**c**) vitamin C (VC_NF) shows a differential rate of erosion, with some regions exhibiting less erosion (white asterisk) and others showing interprismatic enamel loss (white arrow), and (**d**) vitamin C and fluoride (VC_F) show indiscriminate erosion (red arrow), leading to a haphazard enamel surface pattern. (**e**) Salbutamol (SB_NF) shows milder erosive patterns (white arrow), with regions of smooth surface (white asterisk). (**f**) Salbutamol and fluoride (SB_F) show areas of interprismatic enamel loss (white arrow), with a few regions exhibiting indiscriminate erosion (red arrow)

Figure 1(a) shows a photomicrograph of a sound enamel surface stored in distilled water (DW_NF) for 2 weeks, revealing a relatively smooth surface with no marked topographic changes. Figure 1(b) shows a photomicrograph of the enamel surface after exposure to fluoride (DW_F) for 2 weeks, which revealed mild morphological alterations compared with the smoothness of the healthy enamel surface. Slight alterations with protruding enamel rods are evident on enamel surfaces treated with vitamin C (VC_NF) for two weeks, as shown in Fig. 1(c). Figure 1(d) shows greater loss of the morphological surface compared with the other groups, with a considerable amount of dissolution of the prismatic structure depicting enamel after treatment with vitamin C, followed by fluoride application (VC_F). Slight dissolution of the interrod substance of the enamel surface exposed to only salbutamol (SB_NF) can be observed in Fig. 1(e). Raised interrod structures, along with the dissolution of the interprismatic enamel and a few regions of indiscriminate erosion, were observed in the salbutamol and fluoride (SB_F) subgroup, as shown in Fig. 1(f).

## Discussion

The tooth surface constantly maintains a delicate equilibrium between demineralization and remineralization [26]. Disruption of this balance by external acid sources can result in tooth surface loss. One such source of acid may be medications prescribed by doctors [27]. Vitamin C supplements taken long-term, as well as salbutamol inhalers used by asthmatics over extended periods, can have detrimental effects on dental enamel because of their acidic nature [27]. To mitigate this, patients are often advised to use fluoride supplements as a preventive measure [28]. Fluoride formulations have long served as agents for remineralization through the formation of fluorapatite [29]. Some commercially available fluoride mouthwashes can also have an acidic pH. The acidity of the formulation promotes the binding of fluoride to enamel and ensures the stability of fluoride compounds [30]. In this study, samples subjected to vitamin C and anti-asthmatic drugs were subsequently treated with a fluoridated mouthwash to study its remineralization efficacy. This differs from previous investigations, where samples were treated with fluoride prior to acidic exposure to assess its protective effect [31, 32]. This study is the first to investigate the effects of exposure to acidic medications followed by the use of fluoride mouthwash on dental enamel.

The changes in chemical characteristics or mineral loss present postexposure to the medicaments were assessed in the present study via quantitative laser fluorescence. Earlier studies have shown that initial demineralization can be detected via DIAGNOdent^®^ (KaVo), which works on the principle of a noninvasive method involving the use of quantitative laser fluorescence [21, 24, 33]. When the infrared light changes in tooth tissue, such as porosity due to demineralization, it irradiates fluorescent light of a different wavelength, which is detected by a sensor in the device and represented as a numerical value [24, 34, 35]. This method was selected to assess the mineral content of the enamel because of its ease of use and non-destruction of the samples [36].

The baseline QLF values of the enamel samples in this study ranged from 0.5 to 2.63, and after exposure to various agents, the values increased to a range of 1.63–5. As demonstrated in a previous study by Bahrololoomi et al. [24], an increase in DIAGNOdent values indicates greater demineralization, with higher values reflecting more extensive mineral loss. This increase is attributed to the increased porosity of the enamel surface due to a reduction in the overall mineral content [33]. All the subgroups presented a significant increase in the number of QLF readings postexposure, suggesting surface alterations. This could be attributed to the lower pH of the medicaments evaluated in this study. As expected, the control group presented the smallest change in the QLF value (ΔQLF). In contrast, all other subgroups—whether exposed to vitamin C, salbutamol inhalation, or fluoride mouthwash—demonstrated higher ΔQLF values than did the control group (distilled water), indicating increased demineralization. However, these differences were not statistically significant, except in the group exposed to vitamin C, where the ΔQLF value was significantly greater than that of the control group, indicating enhanced enamel demineralization. The long-term use of chewable vitamin C tablets leading to dental erosion is well documented [15]. A compelling case study revealed localized tooth loss in a patient with a three-year history of chewing vitamin C tablets. Upon cessation of tablet consumption, the progression of tooth loss stopped [37]. Our study revealed a similar demineralizing effect on tooth enamel, most likely attributed to its low pH value of 4.5. Thus, the null hypothesis that medications do not influence enamel erosion was rejected.

Surface microhardness is an indirect indicator used to assess the demineralization and remineralization of enamel, with a greater mean difference in hardness indicating a greater degree of enamel demineralization [38]. Previous studies have tested various medications for their demineralizing effects on enamel. For example, Jeong et al. [39] examined the erosive effects of commercially available effervescent vitamins, Gutierrez et al. [14] explored the impact of antiasthmatic inhalers, and Valinoti et al. [40] studied the effects of three acidic pediatric syrups on the surface microhardness of bovine tooth enamel. All these studies demonstrated a significant reduction in the surface microhardness of tooth enamel upon exposure to these agents. However, in the present study, although the tested medications led to a decrease in microhardness, this reduction was not statistically significant compared with that of the control group, except in the group where enamel exposed to vitamin C was immediately treated with fluoride. This difference could be attributed to methodological variations, particularly with respect to pH cycling and the substrate used. In this study, permanent human enamel was used as the substrate, unlike previous studies that employed bovine enamel [14, 39, 40].

This study also examined the remineralization effect of fluoride mouthwash immediately after exposure to acidic medicaments. Research conducted by Molaasadolah et al. [41] and Punathil et al. [42] demonstrated that fluoride treatments significantly increase surface microhardness. In the present study, however, samples exposed to vitamin C alone presented a notable reduction in microhardness, with the most substantial decrease occurring when samples were exposed to both vitamin C and fluoride mouthwash. Notably, after exposure to vitamin C, the samples were immediately submerged in fluoride mouthwash, which could further explain the synergistic effect of the acidic mouthwash combined with the acidic nature of vitamin C. This finding contrasts with the findings of the aforementioned studies but aligns with research conducted by Favaro et al. [14], who reported a decrease in microhardness upon exposure to mouthwashes with lower pH. This contrasting result could be attributed to differences in the timing of fluoride application, the type of fluoride treatment used, and specific study characteristics. The lower fluoride concentration and shorter contact time could also account for the variability in the results. A study by Chedid et al. [43] demonstrated that the use of 0.02% NaF solution (90 µg F/ml) neither reduced enamel demineralization nor improved fluoride uptake by demineralized enamel, highlighting its poor performance in remineralization.

Scanning electron microscopy (SEM) creates high-resolution images of hard surfaces and is therefore an appropriate tool for examining the surface topography of dental enamel. Jeong et al. [39] and Valinoti et al. [40] demonstrated the erosive effects of acidic medications on dental enamel via SEM images. Our study revealed similar erosive patterns associated with the application of acidic medications such as vitamin C tablets and salbutamol. According to the microscopy images, the enamel had a cobblestone appearance, likely caused by chemical erosion, which affected the prism peripheries more than the enamel rods did [25]. As shown in Fig. 1, samples exposed to fluoride after treatment with vitamin C presented the most erosive pattern. This could be related to the established acidic nature of the vitamin C tablets as well as the low pH of the fluoride mouthwash itself. van Swaaij et al. [44] and Delgado et al. [45] reported that many commercially available mouthwashes have a low pH, potentially causing erosive changes on the enamel surface. This study also revealed an erosive effect, potentially caused by the acidity of the fluoride mouthwash. Therefore, commercially available acidic medications, including mouthwashes, should carry a warning or caution for consumers, highlighting their potential to demineralize dental hard tissue [27].

The current study stands out by examining the interaction between fluoride mouthwash and samples treated with vitamin C tablets and salbutamol, with a specific focus on tooth erosion caused by their acidic nature. While previous research has explored erosion caused by acidic medications, ^(4, 10, 14. 25, 40)^ we aimed to delve deeper by investigating the combined effects of these treatments with fluoride mouthwash. In contrast to the anticipated remineralizing effect of fluoride, our findings revealed that acidic properties of the fluoride further demineralized the samples, likely due to the synergistic demineralizing effect of the medications used on the enamel. These findings underscore the need for further research to determine the safest administration practices for acidic medications and to develop effective strategies for mitigating and reversing their erosive effects.

This study has several potential limitations. The administration of medications was restricted to twice-daily doses over a span of two weeks. Notably, these medications are typically prescribed for chronic systemic conditions, often necessitating prolonged usage. Had the evaluation extended to a longer duration, mirroring the customary treatment regimen, it is plausible that the observed demineralization effects would have been even more pronounced [21]. The teeth selected for the study were not from the sample individual or age group and hence could show different susceptibilities to erosion. In this study, all samples were stored in distilled water instead of artificial saliva. The absence of artificial saliva leaves the inherent remineralization properties of saliva unaddressed. Furthermore, artificial saliva adjusted to the pH levels of natural saliva can exhibit buffering capacity under stimulated conditions, a property that distilled water lacks [46]. However, a study by Aykut-Yetkiner et al. [47] highlighted that salivary substitutes can exhibit both remineralizing and demineralizing effects, depending on their composition. To eliminate this variable, artificial saliva was excluded from the present study to focus solely on evaluating the effects of medication on enamel erosion.

Further research is necessary to thoroughly investigate the impact of various commercially available remineralizing agents on enamel when applied immediately after exposure to acidic medications [48–50]. Comparisons between formulations with different pH levels, fluoride concentrations, and active ingredients would provide valuable insights into their mechanisms of action and optimal usage protocols. Additionally, clinical trials investigating the effects of remineralizing agents in patients using asthma medications and other acidic drugs could contribute significant perspectives into the impact of these medications on oral health.

## Conclusion

Chewable vitamin C, salbutamol inhalers, and fluoride mouthwash were found to have pH values below 5.5, indicating their potential to demineralize enamel. Laser fluorescence readings confirmed increased demineralization on enamel surfaces treated with these substances, with the most pronounced effects observed in the vitamin C group. The reduction in microhardness was especially significant in samples exposed to vitamin C followed immediately by acidic fluoride mouthwash. Furthermore, notable alterations in enamel micromorphology were evident in the medication-treated groups, particularly when acidic fluoride mouthwash was applied directly after exposure to the acidic drugs.

## Data Availability

The datasets supporting this study’s findings are not publicly accessible; however, those interested may obtain them by contacting the corresponding author (M.S.T.) upon request.

## Abbreviations

QLF: Quantitative light fluorescence
SEM: Scanning electron microscopy
VHN: Vickers hardness number
SPSS: Statistical Package for the Social Sciences
CEJ: Cementoenamel Junction
DW: Distilled water
VC: Vitamin C
SB: Salbutamol
NF: No fluoride
F: Fluoride
